# Chemical Compositions of Propolis from China and the United States and their Antimicrobial Activities Against *Penicillium notatum*

**DOI:** 10.3390/molecules24193576

**Published:** 2019-10-04

**Authors:** Xiaolan Xu, Ruixue Pu, Yujie Li, Zhenghong Wu, Chunxia Li, Xiaoqing Miao, Wenchao Yang

**Affiliations:** 1 College of Animal Science (College of Bee Science), Fujian Agriculture and Forestry University, Fuzhou 350002, China; xlxufz@fafu.edu.cn (X.X.); wzh516@126.com (Z.W.); 18887266780@139.com (C.L.); mxqsf88@126.com (X.M.); 2 College of Food Science, Fujian Agriculture and Forestry University, Fuzhou 350002, China; puruixue66@126.com (R.P.); 18105041302@163.com (Y.L.); 3 Bee Product Processing and Application Research Center of the Ministry of Education, Fuzhou 350002, China

**Keywords:** propolis, chemical composition, *Penicillium notatum*, antimicrobial activity, iTRAQ

## Abstract

The chemical compositions of ethanol extracts of propolis from China (EEP-C) and the United States (EEP-A) and their antifungal activity against *Penicillium notatum* were determined. The result showed that a total of 49 compounds were detected by UPLC-Q-TOF-MS, 30 of which were present in samples from two regions. The major compounds of EEP-C and EEP-A were similar, including pinocembrin, pinobanksin-3-*O*-acetate, galanin, chrysin, pinobanksin, and pinobanksin-methyl ether, and both of them showed antifungal activity against *P. notatum* with same minimum inhibitory concentration (MIC) value of 0.8 mg·mL^−1^. In the presence of propolis, the mycelial growth was inhibited, the hyphae became shriveled and wrinkled, the extracellular conductivities were increased, and the activities of succinate dehydrogenase (SDH) and malate dehydrogenase (MDH) were decreased. In addition, iTRAQ-based quantitative proteomic analysis of *P. notatum* in response to propolis revealed that a total of 341 proteins were differentially expressed, of which 88 (25.8%) were upregulated and 253 (74.2%) were downregulated. Meanwhile, the differentially expressed proteins (DEPs) involved in energy production and conversion, carbohydrate transport and metabolism, and the sterol biosynthetic pathway were identified. This study revealed that propolis could affect respiration, interfere with energy metabolism, and influence steroid biosynthesis to inhibit the growth of *P. notatum*.

## 1. Introduction

Propolis is a mixture of complex compounds, including flavonoids, phenolic acids, terpenes, coumarins, steroids, amino acids, and mineral elements, which are collected by bees and mixed with its saliva and beeswax [1]. Among them, flavonoids, terpenoids, and phenolic constituents have been reported as the main propolis components that contribute to antimicrobial activity [2,3,4,5,6]. Because the bioactive compound composition of propolis depends on the season, plant species, and the regions of collection, different types of propolis show different chemical composition resulting in different antimicrobial activities against bacterial and fungal pathogens [7,8,9,10,11].

Propolis has broad-spectrum antimicrobial activity against a variety of bacteria and fungi, such as *Escherichia coli*, *Streptococcus mutans,* and *Staphylococcus aureus*, exhibiting great potential for development as an antimicrobial drug without toxicity [2,12,13,14]. Many researchers have explored the antimicrobial activity of propolis. Pinocembrin from propolis could inhibit the respiration of fungi by interfering with energy metabolism and damaging the cell ultrastructure [4,15]. Propolis can induce cell death by regulating the metacaspase and ras signaling pathways, which occur in the transition from yeast-like to hyphal growth [16].

*Penicillium notatum* is a common fungus that not only causes fruit decay but also causes allergies in humans [17,18], but there are few reports on the antifungal activity against *P. notatum*. In the current study, propolis was analyzed for antifungal activities against *P. notatum* in vitro tests. In addition, isobaric tagging for relative and absolute quantitation (iTRAQ) technology, which could be used to analyze the mode of action of the drugs or fungicide against fungal, such as *Aspergillus fumigatus* and *Fusarium graminearum* [19,20], was used to analyze the antifungal activities at the protein level.

## 2. Results

### 2.1. Chemical Composition

The contents of total polyphenols and total flavonoids in propolis are shown in Table 1. The results showed that the content of total flavonoids and phenols of ethanol extracts of propolis from China (EEP-C) were higher than those of ethanol extracts of propolis from United States (EEP-A).

**Figure 1:**
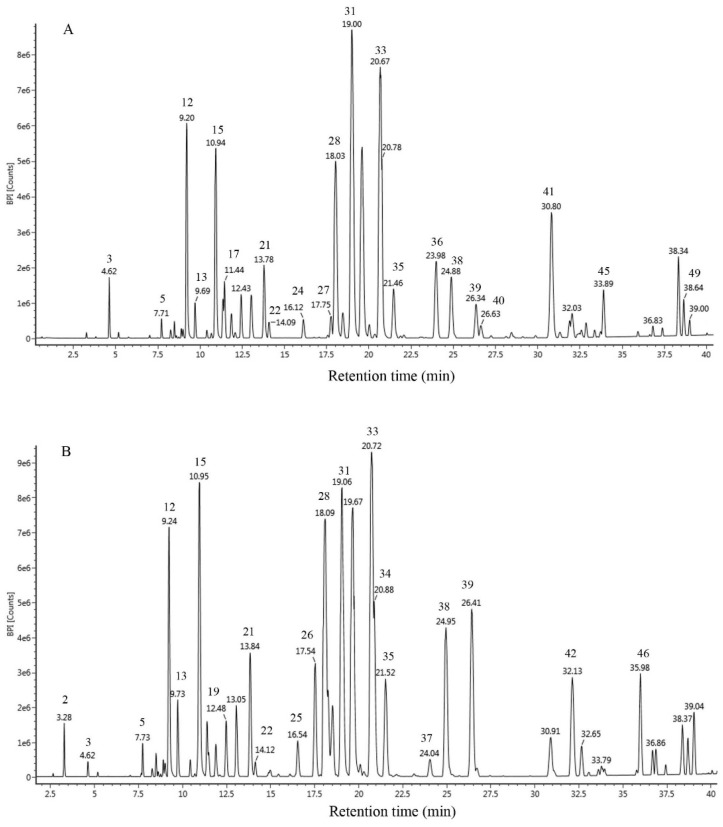
Chromatographic profile of propolis from the United States (**A**) and China (**B**) using UPLC-Q-TOF-MS.

The chromatograms obtained by UPLC-Q-TOF-MS are shown in Figure 1. EEP-A and EEP-C exhibited similar chemical profiles containing a high amount of flavonoids (73.27% in EEP-A and 71.92% in EEP-C). A total of 49 constituents were identified in EEP-A (39) and EEP-C (42), 30 of which were present in two samples (Table 2). Besides, the major components in the two samples were similar, including pinocembrin (16.14% in EEP-A and 9.66% in EEP-C), pinobanksin-3-O-acetate (14.52% in EEP-A and 11.40% in EEP-C), galangin (9.74% in EEP-A and 9.90% in EEP-C), chrysin (8.33% in EEP-A and 9.07% in EEP-C), pinobanksin (5.68% in EEP-A and 6.26% in EEP-C), and pinobanksin-methyl ether (5.36% in EEP-A and 4.24% in EEP-C).

### 2.2. Antifungal Activities

The antifungal effects of EEP against *P. notatum* are shown in Table 3. The values of diameters of inhibition zones showed that EEP-A and EEP-C had antifungal activity. The inhibitory diameter of EEP-A and EEP-C were 8.65 cm to 14.88 cm and 11.79 cm to 16.62 cm, respectively. In addition, their minimum inhibitory concentration (MIC) was the same, both of which were 0.8 mg·mL^−1^.

### 2.3. Effects of EEP on Mycelial Growth

**Figure 2:**
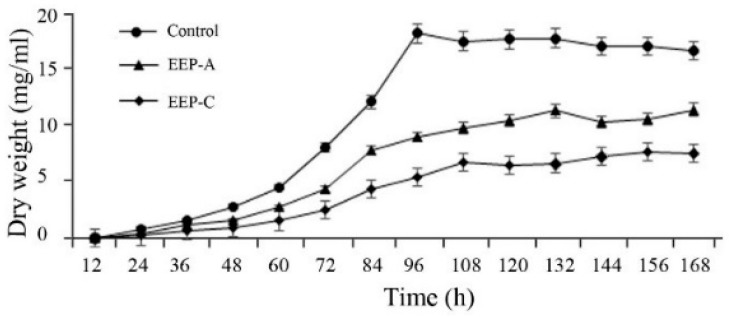
Effect of ethanol extracts of propolis (EEP) on the mycelial growth of *P. notatum.*

As shown in Figure 2, the mycelia of the control group were in the period of slow growth from 0 to 48 h, and in the logarithmic phase from 48 to 96 h, and the dry weight reached a maximum at 96 h. EEP-A and EEP-C treatment significantly inhibited mycelial growth, and EEP-C had a stronger inhibitory effect than EEP-A. According to the results of the growth curve, the mycelia that were cultured for 3 days were used for the following experiments.

**Figure 3:**
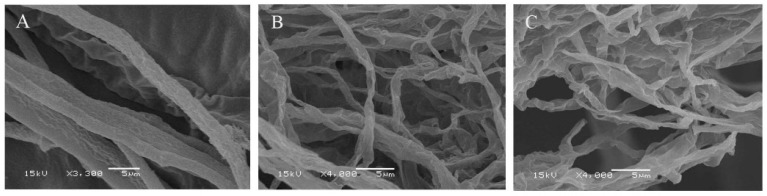
Effect of propolis on the hyphal morphology of *P. notatum.* (**A**) Control, (**B**) ethanol extracts of propolis from United States (EEP-A), (**C**) ethanol extracts of propolis from China (EEP-C).

The morphological changes in hyphae were observed by scanning electron microscopy (Figure 3). The hyphae of the control group were round and full with a smooth surface. While the hyphae of EEP groups were deformed, their surfaces were rough, and the structures were damaged. The results show that propolis can effectively destroy the structure and morphology of hyphae at 1/2 MIC to inhibit the growth of mycelium.

### 2.4. Effect of EEP on the Cell Membrane Permeability

**Figure 4:**
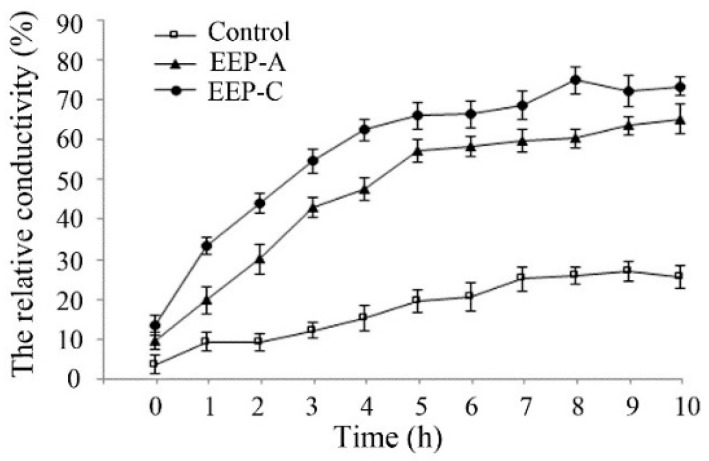
Effect of EEP on the cell membrane permeability of *P. notatum.*

As shown in Figure 4, compared with the control, EEP treatment increased the extracellular conductivity, indicating that EEP could damage the cell membrane.

### 2.5. Effect of EEP on the Activities of Succinate Dehydrogenase (SDH) and Malate Dehydrogenase (MDH)

**Figure 5:**
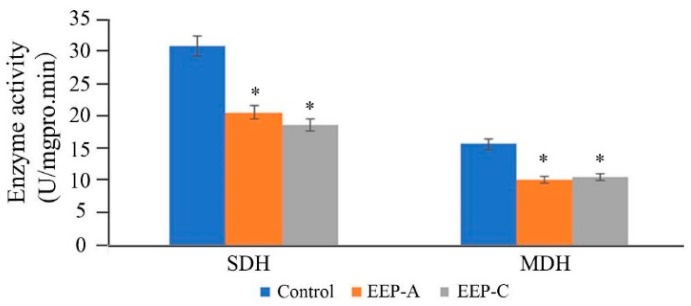
Effect of propolis on succinate dehydrogenase (SDH) and malate dehydrogenase (MDH) activities of *P. notatum* (*n* = 3, * *p* < 0.05).

As shown in Figure 5, SDH and MDH activities of the EEP treatment groups decreased significantly, indicating that propolis could interfere with the respiration of *P. notatum*.

### 2.6. iTRAQ Data Analysis and Differentially Expressed Proteins (DEPs) Identification

EEP-C was used for proteomics experiment; as a result, a total of 341 DEPs were identified in the *P. notatum* proteome in response to propolis, of which 88 (25.8%) were upregulated and 253 (74.2%) were downregulated. According to the Clusters of Orthologous Groups of proteins (COG) functional categories, the DEPs are divided into 23 functional categories (Supplementary Figure S1). Part of the iTRAQ data is listed in Supplementary Table S2, and more detailed information, including protein sequences, can be found in Supplementary Table S3.

### 2.7. RT-PCR Analysis of the Differentially Expressed Proteins

**Figure 6:**
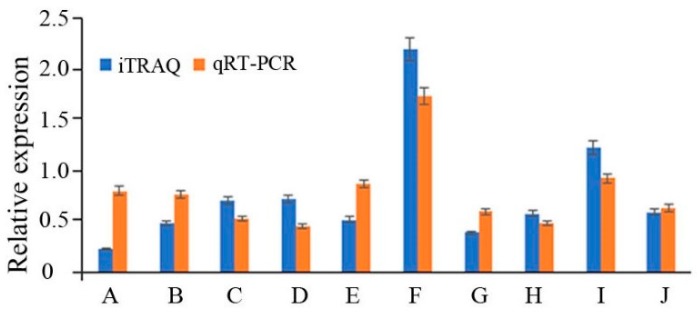
Comparative analysis of RNA and protein levels of differentially expressed proteins. **A**. sterol C-24 reductase (ERG4), **B**. 40S ribosomal protein S8, **C**. translation initiation factor 1A, **D**. zinc finger, **E**. tubulin alpha chain, **F**. acyl-CoA N-acyltransferase, **G**. nicotinamide adenine dinucleotide (NADH) ubiquinone oxidoreductase, **H**. flavin adenine dinucleotide (FAD)-dependent pyridine nucleotide-disulfide oxidoreductase, **I**. ATPase P-type Mg/Cd/Cu/Zn/Na/Ca/Na/H-transporter, **J**. serine/threonine-protein phosphatase.

To validate the reliability of the iTRAQ data, the expression levels of 10 genes were randomly selected for qRT-PCR assays (Figure 6). The results showed that the expression levels detected by qRT-PCR were consistent with the iTRAQ data, indicating that the qRT-PCR data estimate the reliability of the results from the iTRAQ analysis.

## 3. Materials and Methods

### 3.1. Propolis Samples

*P. notatum* AS 3.3871 was purchased from Shanghai Benno Biotechnology Co., Ltd. (Shanghai, China). Propolis from China was supplied by Fujian Shenfeng Technology Development Co., Ltd. (Fuzhou, Fujian, China). Propolis from the United States was collected in Michigan in May 2016.

### 3.2. Preparation of Experimental Samples

Row propolis was extracted using ethanol (70%, *v*/*v*) by the ultrasonic wave method with the parameters of 40 kHz, 20 min, and 60 °C 3 times and then soaked at room temperature for 2 days. The mixture was centrifuged, and the supernatant was partially evaporated under low pressure. After the concentrated propolis extract was stored at 4 °C, beeswax solidified on the surface. This process was repeated three times to remove the beeswax. Finally, the ethanol extract of propolis (EEP) was concentrated under vacuum until the solvent evaporated. The samples were named EEP-C (Chinese propolis) and EEP-A (U.S. propolis).

### 3.3. Total Polyphenol and Total Flavonoid Contents Determination

The Folin–Ciocalteu method was used for the quantification of total polyphenol in each propolis sample [25]. Gallic acid was used as a standard substance. The absorbance of samples was measured at 765 nm, and the total phenolic content was estimated using a calibration curve (Y = 0.0015X − 0.0074, R^2^ = 0.999). The content was expressed in milligram equivalents of gallic acid/gram of dry propolis extract.

The total flavonoid content in the extracts was determined according to the national standards for propolis in China (GB/T 24283-2018). Rutin was used as a standard substance, and the absorbance of samples was measured at 510 nm (Y = 0.005X − 0.004, R^2^ = 0.999).

### 3.4. UPLC-ESI-MS Analysis

EEP (0.2 g) were dissolved with 80% aqueous solution of methanol (20 mL). The extracts were centrifuged at 14,000 rpm for 5 min and diluted 10 times for chemical analyses.

Chemical analyses were performed on a Waters UPLC system using an ACQUITY UPLC HSS T3 column (2.1 × 100 mm, 1.7 μm). The mobile phases A and B consisted of 0.1% formic acid in water and acetonitrile, respectively. The mobile phase gradient program was as follows: 0~8 min, 90% A~70% A; 8~30 min, 70% A~55% A; 30~38 min, 55% A~40% A; 38~42 min, 40% A~30% A; 42~44 min, 30% A~20% A, 44 ~45 min, 20% A ~0 A; 45~47.5 min, 0 A; 47.6 min, 90% A; 47.6~50 min, 90% A. The flow rate was 0.36 mL/min. The on-line UV spectra were recorded at 254 nm, 280 nm, and 330 nm; meanwhile, the samples were scanned from 200 nm to 400 nm.

The mass spectra were acquired in both positive and negative ion modes by using a Waters definition accurate-mass quadrupole time-of-flight (Q-TOF) Xevo G2-XS mass spectrometer (Waters Ltd., Elstree, Hertfordshire, UK) equipped with an ESI source. The optimized operating parameters were as follows: mass range, *m*/*z* 500–1500; the flow rate of drying gas (N2), 800 L/h; drying gas temperature, 400 °C; cone gas flow, 100 L/h; source temperature, 120 °C; capillary voltage, 2.5kV; cone voltage, 40 V. In mass spectrometry (MSE) mode, the energies for collision-induced dissociation (CID) were 6 V for the precursor ion at low energy mode and 30–160 V for fragmentation information at high energy mode. An external reference (Lock-Spray TM) consisting of a 0.2 ng/mL solution of Leucine enkephalin was used in both positive (*m*/*z* 556.2771 [M + H]^+^) and negative mode (*m*/*z* 554.2615 [M − H]^−^), infused at a flow of 5 µL/min. All the data were acquired using Mass Lynx TM 4.1 software (Waters, Milford, MA, USA).

### 3.5. Antifungal Bioassay

In vitro antifungal activity was evaluated by measuring the diameter of the inhibition zone according to Ajay Sharma’s method [26]. The spores were suspended in distilled water containing Tween-80 (0.1%, v/v) to a final density of 1 × 105 CFU/mL and cultivated on a potato dextrose agar (PDA) plate using the spread plate method. An Oxford cup was used to create a well in the agar plate. Then, 50 μL of each EEP solution (0, 20, 40, 60, 80, 100 mg/mL) was added to the wells, and ethanol (70%, v/v) only was added to the wells as a control. After the plates were incubated at 28 °C for 7 days, the diameters of the inhibition zones were measured.

The minimum inhibitory concentration (MIC) values were determined by the agar dilution method. One milliliter of EEP was added to 19 mL of PDA medium, and 5 µL of spore suspension was injected into the center of the solidified medium. PDA without EEP was used as a control. The plates were incubated at 28 °C for 3 days; the concentration that could completely inhibit microbial growth was considered the MIC.

### 3.6. Culture Conditions

The spore suspension was added to potato dextrose broth (PDB) medium at a final concentration of 1 × 10^5^ CFU·mL^−1^, EEPs were added to the medium at a final concentration of 1/2 MIC, and ethanol (70%, *v*/*v*) was added to the medium as a control. The mycelia were incubated at 28 °C and 180 rpm for 7 days, and the dry weight of the mycelia was weighed every 12 h to evaluate the effect of propolis on mycelial growth. The mycelia in the logarithmic growth period were used in the following experiments.

### 3.7. Scanning Electron Microscope (SEM) Observation

The hyphae were fixed with 5% glutaric dialdehyde and 1% osmic acid for 4 h. After treatment with 50%, 70%, 80%, 90%, and 100% alcohol for dehydration, the hyphae were observed under SEM by JSM-6380LV (JEOL Co., Tokyo, Japan).

### 3.8. Effects of EEP on mycelia Cell Membrane Permeability

The mycelia were placed in 100 mL of sterile water. Then, EEPs were added to the water at a final concentration of 1/2 MIC. The different treatment groups were cultured at 28 °C, and the electrical conductivities of the EEP treatment group (Pn) and control group (P0) were determined once every 1 h. The treatment group was boiled for 10 min and cooled to determine the electrical conductivity of the mycelium (Pk). An equal concentration of ethanol was used as a control. The electrical conductivity was calculated using the following formula:

The electrical conductivity(%)=(Pn−P0)/(Pk−P0)×100%

### 3.9. Enzyme Activity Assays

The effects of EEP on SDH and MDH were determined. The mycelia were ground in phosphate buffer (0.1 mol·L^−1^, pH 7.5). After centrifugation, the supernatants were collected to detect the enzyme activity according to the procedure of the enzyme reagent kits (Nanjing Jiancheng Bioeng. Inst., Nanjing, Jiangsu, China), and the protein content of the hyphae was determined using the Bradford method (Nanjing Jiancheng Bioeng. Inst., Nanjing, Jiangsu, China). The enzyme activities were expressed as units per milligram of protein.

### 3.10. iTRAQ Experiment

The spore suspension was added to PDA liquid medium to yield a final density of 1 × 10^6^ FU/mL, and then EEPs were added to obtain a final concentration of 1/2 MIC. An equal volume of ethanol (70%, *v*/*v*) instead of EEP was used as a control. The fungus was incubated at 28 °C for 3 days, and the hyphae were suspended in lysis buffer (7 M urea; 2 M thiourea; 4% CHAPS; 40 mM Tris-HCl, pH 8.5; 1 mM PMSF; 2 mM EDTA) and sonicated on ice. The proteins were reduced with 10 mM DTT at 56 °C for 1 h and then alkylated by 55 mM iodoacetamide (IAM) in a dark room for 1 h. The reduced and alkylated protein mixtures were precipitated by adding 4 × volume of chilled acetone at −20 °C overnight. After centrifugation at 4 °C and 30,000× *g*, the pellet was dissolved in 0.5 M triethylammonium bicarbonate (TEAB) (Applied Biosystems, Milan, Italy) and sonicated on ice. After centrifuging at 30,000× *g* at 4 °C, an aliquot of the supernatant was taken to determine protein concentration. The proteins in the supernatant were kept at −80 °C for further analysis.

Total protein (100 μg) was removed from each sample, and then the protein was digested with Trypsin Gold (Promega, Madison, WI, USA). Peptides were processed according to the manufacturer’s protocol for 8-plex iTRAQ reagent (Applied Biosystems, Milan, Italy). SCX chromatography was performed with an LC-20AB HPLC pump system (Shimadzu, Kyoto, Japan). Data acquisition was performed with a TripleTOF 5600 System (AB SCIEX, Concord, ON, Canada) fitted with a Nanospray III source (AB SCIEX, Concord, ON, Canada) and a pulled quartz tip as the emitter (New Objectives, Woburn, MA, USA).

The iTRAQ data were analyzed using Mascot software (version 2.3.02, Matrix Science Inc., Boston, MA, USA). Functional annotations of the proteins were conducted using the Blast2GO program against the non-redundant protein database (NR, NCBI). The Kyoto Encyclopedia of Genes and Genomes (KEGG) database and the KOG database (NCBI) were used to classify and group the identified proteins.

### 3.11. Quantitative Real-Time PCR

Total RNA was extracted using Trizol reagent (Invitrogen, Carlsbad, CA, USA). cDNA synthesis was performed using the PrimeScript^TM^ RT reagent kit with gDNA Eraser (TaKaRa). qPCR was performed using the SYBR^®^ Premix ExTaq^TM^ kit (TaKaRa). The PCR primers were designed using primer software 6.0, and the sequences are listed in Supplementary Table S1. PCRs were performed using the Applied Biosystems 7500 Real-Time PCR System. The cycling conditions were 94 °C for 3 min followed by 40 cycles at 94 °C for 30 s and 60 °C for 34 s. To standardize the target gene level with respect to variability in the quality of RNA and cDNA, GAPDH was amplified under the same conditions as an internal control.

### 3.12. Statistical Analysis

All experiments were performed in triplicate. The data are reported as the mean (*n* = 3) ± standard deviation. The differences among groups were tested by paired-samples T-test of SPSS software (IBM^®^ SPSS^®^ Statistics, versus 19.0). Differences of *p* < 0.05 were considered statistically significant.

## 4. Discussion

In this study, the concentrations of total phenols, flavonoids, and chemical composition of propolis, as well as their antifungal activities, were determined, and propolis from China and the United States had an indifferent chemical composition and antifungal activities. It has been reported that the antifungal effect of propolis extract is mainly attributed to the flavonoid and phenolic components [3,27], especially chrysin, galanin, cinnamic acid, caffeic acid, and their derivatives [28,29,30,31,32]. In this study, the major compounds of both regions were pinocembrin, galanin, chrysin, etc., and the results were consistent with previous studies of polar-type propolis [33,34]. So far, no studies have reported the composition of propolis from Michigan. It has been demonstrated that the propolis component is dependent on plant sources [34]. Polar tree *(Populus spp.*) is found in North America and China, and EEP-A showed similar peaks with EEP-C, indicating it also belongs to polar-type propolis. When the MIC value was in the range of 100–1000 mg mL^−1^ in vitro susceptibility tests, the propolis was thought to have antifungal properties [35,36]. The results revealed that EEP-A and EEP-C showed a potent effect against *P. notatum.*

EEP-A and EEP-C can inhibit the growth of mycelia, change the morphology of mycelia, and affect the relative permeability of mycelial cell membranes to destroy the formation of biofilms [37,38]. In the current study, most proteins related to mycelial growth, such as amino acid metabolism, translation, ribosomal structure, and biogenesis, were downregulated in the presence of propolis (Supplementary Table S3).

Of the 23 proteins involved in amino acid transport and metabolism, 18 were downregulated, and five were upregulated (Supplementary Table S2). Among them, tryptophan synthase, glutamine synthetase, and methionine synthase, which directly participate in the synthesis of amino acids, were downregulated, indicating that EEP can affect the synthesis and metabolism of amino acids. Of the 32 proteins involved in translation, ribosomal structure, and biogenesis, 24 were downregulated, and eight were upregulated. Among the 13 ribosomal proteins, only two were upregulated, and the others were downregulated. Ribosomal proteins and ribosomal RNA constitute the ribosome, play important roles in ribosomal assembly and protein synthesis [39], and also have many extrachromosomal functions, including DNA damage repair, gene expression regulation, mRNA translation, cell proliferation, differentiation, and apoptosis. Ribosomal protein deficiency can cause cell cycle arrest and apoptosis [40,41,42]. Amino acids are protein precursors, and ribosomes are the sites of protein synthesis; thus, the downregulation of the expression of these proteins also affects the synthesis of proteins, resulting in a decrease in the amount of the total protein.

EEP can reduce the activities of MDH and SDH, which are related to energy metabolism processes, including the tricarboxylic acid cycle and oxidative phosphorylation. In eukaryotic mitochondria, MDH and SDH are the key enzymes of glucose metabolism and the tricarboxylic acid cycle, respectively, which can be used as indicators of respiratory metabolism. Inhibition of the activity of microbial respiratory enzymes is one of the main mechanisms of some drugs against pathogenic fungi [43,44,45,46]. Yao et al. found that extracts of nobiletin and tangeretin could strongly inhibit the activities of SDH and MDH in *Pseudomonas* to inhibit the growth of mycelia [37].

In the proteome experiment, there were 25 proteins related to energy metabolism: three were upregulated, and the other 22 were downregulated (Supplementary Table S2). Among them, some proteins involved in the respiratory electron transport chain, including cytochrome c oxidase subunit 6A, nicotinamide adenine dinucleotide (NADH) dehydrogenase, NADH-ubiquinone oxidoreductase, and flavin adenine dinucleotide (FAD)/flavin Mononucleotide (FMN)-containing dehydrogenases, were downregulated after EEP treatment. Because the mitochondrial respiratory chain relates to energy metabolism, the inhibition of respiration would interfere with many physiological processes [47,48,49,50]. Currently, the respiratory chain in pathogenic fungi has been proposed as a potential antimicrobial target [51]. For example, antimycin A could bind to cytochrome c reductase in mitochondrial complex III and block the mitochondrial electron transfer between cytochrome b and c to inhibit cell growth [52,53]. Inhibitors of the respiratory chain were also efficient in blocking the germination of *A. fumigatus* by inhibiting protein synthesis [54].

The inhibition of the respiratory chain would lead to a decrease in the cellular levels of ATP production and cause cell death [55,56]. In this study, proteins that are involved in the tricarboxylic acid cycle (TCA cycle) were downregulated, including isocitrate dehydrogenase (IDH), succinyl-CoA synthetase, and pyruvate dehydrogenase complex (PDHC) (Supplementary Table S2). TCA is the common pathway through which sugars, proteins, and fats are completely oxidized to produce ATP. Moreover, the intermediate products in this cycle (such as oxaloacetic acid and α-ketoglutaric acid) are materials for the synthesis of sugar, amino acids, fats, etc. As a result, the TCA cycle exhibited a strong correlation with fungal growth [57,58]. IDH catalyzes isocitrate to produce ketoglutarate, NADH, and CO_2_. This reaction is a rate-limiting and irreversible step. Succinyl CoA synthetase could catalyze succinyl CoA to generate succinic acid and generate GTP. These two enzymes affect the production of ATP and thus interfere with energy metabolism. Besides, PDHC connects the aerobic oxidation of sugar with the TCA cycle and oxidative phosphorylation and plays a crucial role in the energy metabolism of the mitochondrial respiratory chain in cells [59]. The above results suggested that the respiration and energy metabolism of *P. notatum* interfered in response to EEP.

Besides, ergosterol, a fungus-specific sterol, enriched in cell plasma membranes, is an effective antifungal drug target. Amiodarone, fluconazole, naftifine hydrochloride, and terbinafine could inhibit the growth of mycelia by inhibiting the key enzymes in the synthetic pathway of sterol and interfering with the biosynthesis of ergosterol [60,61,62]. In this study, proteome analysis indicated that EEP could downregulate the *ERG4* and *ERG9* genes of the ergosterol biosynthesis pathway (Supplementary Table S2). The *ERG4* gene encodes the sterol C-24 reductase, which catalyzes the conversion of ergosta-5,7,22,24-tetraenol to ergosterol in the final step of ergosterol biosynthesis. In *Fusarium graminearum*, *ERG4* (*FgERG4*) deletion mutant could not synthesize ergosterol, resulting in a significant decrease in mycelial growth and conidiation and abnormal conidia production [63,64]. ERG9 (squalene synthase) is the first committed enzyme of the sterol biosynthesis pathway. The strain overexpressing the gene *ERG9* also displayed significant inhibition of growth in the presence of ferrozine, calcium deprivation, and osmotic/ionic stress [65]. ERG4 and ERG9 play an important role in the process of sterol synthesis, and inhibitors of these enzymes have been intensively studied as potential antifungal agents; thus, their downregulation suggested that the synthesis of sterols of *P. notatum* were downregulated in response to EEP.

## 5. Conclusions

In this work, UPLC-Q-TOF-MS method was used to analyze the chemical compositions of ethanol extracts of propolis from China and the United States. The results showed that the propolis from two regions had a high content of flavonoids, which exhibited broad-spectrum antimicrobial activity against a variety of bacteria and fungi. In vitro test of EEP against *P. notatum*, EEP could inhibit the mycelial growth, destroy the hyphae structure and permeability of the cell membrane, and decrease the activities of succinate dehydrogenase (SDH) and malate dehydrogenase (MDH). Meanwhile, DEPs involved in energy production and conversion, carbohydrate transport and metabolism, and the sterol biosynthetic pathway were also identified. This study revealed that propolis could affect respiration, interfere with energy metabolism, and influence steroid biosynthesis to inhibit the growth of *P. notatum*.

## Figures and Tables

**Table 1:** Total phenol and flavonoid contents of propolis and bud poplar resins.

Samples	Total Phenol (mg·g^−1^)	Total Flavonoids (mg·g^−1^)
EEP-A	370.19 ± 4.12b	197.93 ± 1.24c
EEP-C	440.18 ± 5.11a	305.60 ± 1.69b

EEP-A: ethanol extracts of propolis from United States, EEP-C: ethanol extracts of propolis from China. Note: different letters in the same column indicate significant differences between groups (*p* < 0.05).

**Table 2:** Main components of EEP-A and EEP-C.

No	tR (min)	λmax (nm)	Selected Ion	Formula	Measured Mass	Calculated Mass	Mass Error (MD)	MS/MS Fragmentation	Compound Name	Relative Area(%)
1	2.66	238, 281	[M − H]	C_7_H_6_O_3_	137.0245	137.0239	0.6	137.0245	*p*-Hydroxybenzoic acid ^(b)^	0.02% (C)
2	3.29	243, 324	[M − H]	C_9_H_8_O_4_	179.0351	179.0344	0.7	179.0344, 161.0243, 135.0448	Caffeic acid ^(b, c)^	0.06% (A), 0.45% (C)
3	4.61	237, 310	[M − H]	C_9_H_8_O_3_	163.0400	163.0395	0.5	163.0400, 119.0502, 93.0368	*p*-Coumaric acid ^(b, c)^	1.31% (A), 0.15% (C)
4	5.20	243,323	[M − H]	C_10_H_10_O_4_	193.0505	193.0505	0	193.0505,133.0291	Isoferulic acid ^(b, c)^	0.04% (C)
5	7.71	265, 320	[M − H]	C_21_H_20_O_10_	431.1013	431.0978	3.5	431.1013, 268.0394	Apigenin-7-*O*-β-D-glucopyranoside ^(b)^	0.27% (A), 0.35% (C)
6	8.29	250, 365	[M − H]	C_16_H_11_O_6_	299.0581	299.0556	2.5	299.0581, 227.0363, 129.0349	Kaempferol-methyl ether ^(b)^	0.13% (A), 0.09% (C)
7	8.51	292	[M − H]	C_16_H_14_O_5_	285.0783	285.0763	2.0	285.0783, 267.0668, 252.0437, 239.0722, 138.0326	Pinobanksin-methyl ether ^(b)^	0.24% (C)
8	8.63	250, 365	[M − H]	C_17_H_14_O_7_	329.0683	329.0661	2.4	329.0683, 314.0441, 299.0209, 271.0246, 135.0458	Quercetin-dimethyl ether ^(b)^	0.03% (A), 0.05% (C)
9	8.92	292	[M − H]	C_16_H_14_O_5_	285.0779	285.0763	1.6	269.04620, 139.0406, 124.0170	Pinobanksin-5-methyl ether ^(b, c)^	0.13% (A), 0.15% (C)
10	8.92	251, 349	[M − H]	C_15_H_10_O_6_	285.0418	285.0399	1.9	269.0462, 151.0039	Luteolin ^(a, b)^	0.13% (A), 0.15% (C)
11	9.02	253, 372	[M − H]	C_15_H_10_O_7_	301.0365	301.0348	1.7	243.02886, 151.00304	Quercetin ^(a, b)^	0.15% (A), 0.17% (C)
12	9.20	287	[M − H]	C_16_H_14_O_5_	285.0778	285.0763	1.5	285.0778, 267.0667, 252.0432, 239.0716, 138.0325	Pinobanksin-methyl ether isomer ^(b)^	5.36% (A), 4.24% (C)
13	9.74	255, 352	[M − H]	C_16_H_11_O_7_	315.0514	315.0505	0.9	315.0514, 300.0277	Quercetin-3-methyl ether ^(b, c)^	0.61% (A), 1.00% (C)
14	10.44	265, 312	[M − H]	C_16_H_12_O_4_	267.0663	267.0657	0.6	252.0424, 224.0476	Chrysin-5-methyl ether ^(b, c)^	0.74% (A), 0.21% (C)
15	10.94	292	[M − H]	C_15_H_12_O_5_	271.0613	271.0606	0.7	271.0613, 253.0504	Pinobanksin ^(b, c)^	5.68% (A), 6.26% (C)
16	11.40	265, 365	[M − H]	C_15_H_10_O_6_	285.0400	285.0399	0.1	285.0400, 227.03402	Kaempferol ^(a, b)^	0.74% (A), 0.21% (C)
17	11.44	285	[M − H]	C_16_H_14_O_4_	269.0813	269.0814	−0.1	269.0813, 254.0571, 227.0701, 165.0186	Pinocembrin-5-methyl ether ^(b, c)^	1.22% (A), 0.26% (C)
18	11.90	251, 366	[M − H]	C_16_H_11_O_7_	315.0504	315.0505	−0.1	315.05045, 300.02677, 151.00311	Isorhamnetin ^(b, c)^	0.60% (A), 0.53% (C)
19	12.43	266, 349	[M − H]	C_16_H_11_O_6_	299.0555	299.0556	−0.1	299.0555, 284.03186	Kaempferol-3-methyl ether ^(b, c)^	1.08% (A), 0.99% (C)
20	13.06	251, 351	[M − H]	C_17_H_14_O_7_	329.0664	329.0661	0.3	329.0664, 314.0426, 299.0193, 271.0246, 133.0291	Quercetin-dimethyl ether isomer ^(b, c)^	1.30% (A), 1.29% (C);
21	13.85	259, 350	[M − H]	C_16_H_12_O_5_	283.0618	283.0606	1.2	283.0618, 268.0379, 239.0352	Galangin-5-methyl ether ^(b, c)^	2.10% (A), 2.56% (C)
22	14.13	292	[M − H]	C_18_H_16_O_6_	327.0880	327.0869	1.1	285.0768, 252.0429	Pinobanksin-5-methyl ether-3-*O*-acetate ^(b, c)^	0.41% (A), 0.26% (C)
23	14.94	251, 365	[M − H]	C_16_H_11_O_6_	299.0557	299.0556	0.1	299.0557, 284.0322, 151.0027	Kaempferol-7-methyl ether ^(b)^	0.11% (C)
24	16.12	251, 366	[M − H]	C_17_H_14_O_7_	329.0665	329.0661	0.4	329.0660, 314.0411, 299.0186, 271.0246, 161.0235	Quercetin-dimethyl ether isomer ^(b)^	0.57% (A)
25	16.55	251,351	[M − H]	C_17_H_14_O_7_	329.0664	329.0661	0.3	329.0664, 314.0426, 299.0193, 271.0246, 133.0292	Quercetin-dimethyl ether isomer ^(b)^	0.79% (C)
26	17.53	324	[M − H]	C_14_H_16_O_4_	247.0971	247.0970	0.1	247.0971, 179.0341, 161.0236, 135.0443	Caffeic acid isoprenyl ester ^(b, c)^	2.75% (C),
27	17.75	251,350	[M − H]	C_18_H_16_O_7_	343.0818	343.0818	0	343.0818, 328.0579, 313.0345, 298.0113	Quercetin-trimethyl ether ^(b)^	0.69% (A)
28	18.03	267, 313	[M − H]	C_15_H_10_O_4_	253.0511	253.0501	1.0	145.0294, 107.0138	Chrysin ^(a, b)^	8.33% (A), 9.07% (C)
29	18.25	324	[M − H]	C_14_H_16_O_4_	247.0977	247.0970	0.7	179.0346, 161.0241, 135.0449	Caffeic acid isoprenyl ester isomer ^(b, c)^	1.48% (C)
30	18.52	324	[M − H]	C_16_H_14_O_4_	269.0821	269.0814	0.7	269.0821, 179.9343m 133.0294	Caffeic acid benzyl ester ^(b, c)^	0.88% (A), 1.91% (C)
31	19.00	286	[M − H]	C_15_H_12_O_4_	255.0662	255.0657	0.5	213.0552, 151.0031, 107.0134	Pinocembrin ^(b, c)^	16.14% (A), 9.66% (C)
32	19.65	265, 361	[M − H]	C_15_H_10_O_5_	269.0456	269.0450	0.6	211.03914, 145.0288, 117.0340	Galangin ^(a, b)^	9.74% (A), 9.90% (C)
33	20.67	292	[M − H]	C_17_H_14_O_6_	313.0745	313.0712	3.3	253.0505, 119.0498	Pinobanksin-3-*O*-acetate ^(b, c)^	14.52% (A), 11.40% (C)
34	20.88	324	[M − H]	C_17_H_16_O_4_	283.0975	283.0970	0.5	179.0347, 161.0240, 135.0447	Phenethyl caffeate ^(b,c)^	2.96% (C)
35	21.46	265, 323	[M − H]	C_16_H_12_O_5_	283.0611	283.0606	0.5	283.0611, 268.03733, 239.0346	Acacetin ^(b, c)^	2.00% (A), 2.77% (C)
36	23.98	310	[M − H]	C_16_H_14_O_3_	253.0866	253.0865	0.1	145.0287, 117.0339	*p*-Coumaric acid benzyl ester ^(b,d)^	3.46% (A)
37	24.08	292	[M − H]	C_22_H_22_O_8_	461.1248	461.1236	1.2	401.1012, 253.0874	Pinobanksin-3-*O*-acetate -5-*O*-phydroxyphenylpropionate ^(b,c)^	0.48% (C)
38	24.88	324	[M − H]	C_18_H_16_O_4_	295.0984	295.0970	1.4	178.0270, 133.0296	Caffeic acid cinnamyl ester ^(b, c)^	2.63% (A), 5.00% (C)
39	26.42	292	[M − H]	C_18_H_16_O_6_	327.0874	327.0869	0.5	271.0604, 253.0504	Pinobanksin-3-*O*-propionate ^(b, c)^	1.41% (A), 5.50% (C)
40	26.34	310	[M − H]	C_18_H_16_O_3_	267.1021	267.1021	0	163.0390,145.0288, 119.0495	*p*-Coumaric acid benylethyl ester ^(b)^	0.51% (A)
41	30.80	310	[M − H]	C_18_H_16_O_3_	279.1027	279.1021	0.6	235.1120, 134.0355, 163.0028	*p*-Coumaric cinnamyl ester ^(b,d)^	6.25% (A)
42	32.13	292	[M − H]	C_19_H_18_O_6_	341.1036	341.1025	1.1	271.0610,253.0509	Pinobanksin-3-*O*-butyrate ^(b,c)^	3.23% (C)
43	32.65	292	[M − H]	C_20_H_18_O_6_	353.1039	353.1025	1.4	297.1532, 253.0511	Pinobanksin-3-*O*--pentenoate ^(b,c)^	0.72% (A)
44	33.38	292	[M − H]	C_27_H_24_O_8_	475.1397	475.1393	0.4	415.1174, 264.0500, 134.0366	Pinobanksin-3-*O*-acetate-5-*O*-phydroxyphenylpropionate ^(b,c)^	0.18% (A)
45	33.89	292	[M − H]	C_22_H_16_O_6_	375.0894	375.0869	2.5	271.0603,253.0504	Pinobanksin-3-*O*-benzoate ^(b)^	1.53% (A)
46	35.98	292	[M − H]	C_20_H_20_O_6_	355.1207	355.1182	2.5	253.0520, 271.0619	Pinobanksin-3-*O*-pentanoate or 2-methylbutyrateb ^(b, c)^	0.13% (A), 2.85% (C)
47	36.68	292	[M − H]	C_21_H_20_O_6_	367.1205	367.1182	2.3	271.0624,253.0512	Pinobanksin-*O*-hexenoate ^b,c^	0.56% (C)
48	37.43	292	[M − H]	C_24_H_20_O_6_	403.1200	403.1182	1.8	297.1132, 271.0614, 253.0513	Pinobanksin-3-*O*-phenylpropionate ^(b, c)^	0.20% (A), 0.19% (C)
49	38.69	292	[M − H]	C_21_H_22_O_6_	369.1352	369.1338	1.4	271.0612, 253.0511	Pinobanksin-3-*O*-hexanoate ^(b, c)^	1.08% (A), 0.84% (C)

tR(min): Retention time (min); λmax: the maximum absorption wavelength. ^a^ Confirmed with the standard; ^b^ confirmed with MS fragmentation; ^c^ confirmed with references [21]; ^d^ confirmed with references [22]; ^e^ confirmed with references [23]; ^f^ confirmed with references [24].

**Table 3:** The diameters of inhibition zones.

	Concentration (mg·mL^−1^)					MIC (mg·mL^−1^)
	20	40	60	80	100	
EEP-A	8.65 ± 0.83h	10.33 ± 1.04g	12.09 ± 0.95e	13.66 ± 1.04d	14.88 ± 2.05c	0.8
EEP-C	11.79 ± 1.09e	13.74 ± 0.04d	14.28 ± 2.11d	15.16 ± 0.94b	16.62 ± 1.77a	0.8
70% ethanol	7.04 ± 0.14					-

MIC: minimum inhibitory concentration. Note: different letters indicate significant differences between groups (*p* < 0.05).

## Supplementary Materials

The following are available online. Supplementary Table S1. Primer pairs used for qRT-PCR expression analysis; Supplementary Table S2. Partial DEPs of *P. notatum* in response to EEP; Supplementary Table S3. DEPs of *P. notatum* in response to propolis; Supplementary Figure S1. COG functional categories of DEPs.
