# Supplementary material for: Chemical Compositions of Propolis from China and the United States and their Antimicrobial Activities Against Penicillium notatum
Source: Molecules. 2019 Oct 4;24(19):3576. doi: 10.3390/molecules24193576 (PMC6803850; doi:10.3390/molecules24193576)
Supplement: Supplementary file 1 [file molecules-24-03576-s001.zip › Supplementary table 1 and Supplementary table 2.docx]

**Supplementary table 1.** **Primer pairs used for qRT-PCR expression analysis**

| Gene ID | Forward primers (5′- 3′) | Reverse primers (5′- 3′) |
| --- | --- | --- |
| 1 | GAAGAGTGCGACCTATGA | CGAGATACCAGCCTTGATA |
| 2 | CGACTTTGTGGTGTGGACAT | CAAGCCTGGTCTCATCTCCA |
| 3 | AGAAGTTCCGTGGTCTCCG | GTGAGCCTCGTACCACTGAC |
| 4 | TCTGCTTTGATGGCGAGAAG | CGTAAGCCTTCAGAGAACGG |
| 5 | CCATCCTACAACCCTTCGAG | GAAACTCGCTTCGGACAGTG |
| 6 | TGTTCCACCCAGAGCAATTG | CCACGCTAGGAGACGGATAG |
| 7 | AACAGACTACACGATCCGGC | GCAGATCACGAGCATGTAATACT |
| 8 | CCGCACCAAGGACATGATTT | CTTGCAAGTACCGGGCTCA |
| 9 | CCCGCCTGACCCATCGAA | AGCAAGGGAGTGAACAGGAA |
| 10 | CACGATGAGCCAGAACCCAT | ACGACTTCTCCGACCGATAC |
| 11 | CACAGGTTTTGAAGGCCGAG | CCCAGAGATAGAGGACGCAC |

1.GAPDH, 2. ERG4, 3. 40S ribosomal protein S8, 4. translation initiation factor 1A, 5. zinc finger, 6. tubulin alpha chain, 7. Acyl-CoA N-acyltransferase, 8. NADH ubiquinone oxidoreductase, 9. FAD-dependent pyridine nucleotide-disulfide oxidoreductase, 10. ATPase P-type K/Mg/Cd/Cu/Zn/Na/Ca/Na/H-transporter, 11. serine/threonine-protein phosphatase

**Supplementary table 2.** **DEPs of *P. notatum* in response to EEP**

| **Protein** | **Protein_ID** | **NCBInr Identity** | **NCBInr E-value** | **EEP-VS-CK** |
| --- | --- | --- | --- | --- |
| Amino acid transport and metabolism |  |  |  |  |
| Tryptophan synthase | tr\|A0A0A2KWA4 | 97.93 | 0 | 0.56 |
| Glutamine synthetase | tr\|A0A0A2KBA1 | 99.16 | 0 | 0.46 |
| Methionine synthase | tr\|A0A0A2KQY7 | 95.27 | 0 | 0.78 |
| Energy production and conversion |  |  |  |  |
| Cytochrome c oxidase subunit 6A, | tr\|A0A0A2KU38 | 98.57 | 3.00E-102 | 0.5 |
| NADH dehydrogenase, subunit C | tr\|A0A0A2KY38 | 95.68 | 0 | 0.6 |
| NADH-ubiquinone oxidoreductase | tr\|A0A0A2L206 | 95.22 | 2.00E-150 | 0.53 |
| NADH ubiquinone oxidoreductase, F subunit | tr\|A0A0A2LQ81 | 99.8 | 0 | 0.39 |
| Succinate CoA transferase | tr\|A0A0A2LDY5 | 97.33 | 0 | 2.74 |
| Isocitrate dehydrogenase [NAD] subunit | tr\|A0A0A2L9B4 | 100 | 0 | 2.05 |
| NADPH--cytochrome P450 reductase | tr\|A0A0A2LIN5 | 95.13 | 0 | 0.52 |
| Succinyl-CoA synthetase, alpha subunit | tr\|A0A0A2L0N4 | 95.05 | 0 | 0.46 |
| Succinyl-CoA synthetase, beta subunit | tr\|A0A0A2LAY6 | 98.56 | 0 | 0.81 |
| Succinyl-CoA synthetase, alpha subunit | tr\|A0A0A2L8D0 | 99.39 | 0 | 0.53 |
| NAD-dependent aldehyde dehydrogenases | tr\|A0A0A2KND7 | 96.81 | 0 | 0.5 |
| NAD-dependent aldehyde dehydrogenases | tr\|A0A0A2KNC3 | 96.52 | 0 | 0.48 |
| NAD-dependent aldehyde dehydrogenases | tr\|A0A0A2KRP0 | 93.03 | 0 | 0.54 |
| NAD-dependent aldehyde dehydrogenases | tr\|A0A0A2L2C8 | 88.78 | 0 | 0.59 |
| FAD/FMN-containing dehydrogenases | tr\|A0A0A2K6V7 | 97.97 | 0 | 0.62 |
| FAD/FMN-containing dehydrogenases | tr\|A0A0A2LD52 | 95.26 | 0 | 0.78 |
| **Translation, ribosomal structure and biogenesis** | | | | |
| Transcription elongation factor Spt6 | tr\|A0A0A2KEU9 | 95.58 | 0 | 1.39 |
| 40S ribosomal protein S8 | tr\|A0A0A2KJQ1 | 97.5 | 5.00E-133 | 0.48 |
| Ribosomal protein S27a | tr\|A0A0A2L8P2 | 99.35 | 4.00E-82 | 0.63 |
| Translation elongation factor | tr\|A0A0A2LA58 | 94.77 | 0 | 0.68 |
| Translation elongation factor | tr\|A0A0A2KD71 | 97.17 | 0 | 0.53 |
| Ribosomal protein L4/L1e | tr\|A0A0A2L599 | 99.19 | 0 | 0.36 |
| Ribosomal protein S11 | tr\|A0A0A2L4M8 | 93.14 | 0 | 0.64 |
| Translation Initiation factor eIF-4e | tr\|A0A0A2LDR6 | 97.24 | 0 | 0.43 |
| 60S ribosomal protein L27 | tr\|A0A0A2L9M6 | 98.52 | 4.00E-94 | 0.61 |
| Translation initiation factor 1A | tr\|A0A0A2KMM5 | 99.19 | 4.00E-84 | 0.71 |
| Ribosomal protein S10 | tr\|A0A0A2K739 | 93.58 | 2.00E-125 | 0.39 |
| Ribosomal protein L7A/L8 | tr\|A0A0A2L9L1 | 97.74 | 2.00E-179 | 0.4 |
| Ribosomal protein S13 | tr\|A0A0A2LCB3 | 100 | 4.00E-97 | 0.2 |
| Ribosomal protein L22 | tr\|A0A0A2KPY0 | 94.93 | 0 | 0.83 |
| Elongation factor 1-alpha | tr\|A0A0A2L9N0 | 99.32 | 0 | 0.58 |
| Ribosomal protein S17e | tr\|A0A0A2LCN0 | 100 | 2.00E-89 | 0.4 |
| 60S ribosomal protein L20 | tr\|A0A0A2KH01 | 99.44 | 6.00E-132 | 1.67 |
| Ribosomal protein S26e | tr\|A0A0A2L9A2 | 100 | 3.00E-61 | 1.91 |
| 40S ribosomal protein S21 | tr\|A0A0A2LLL6 | 98.86 | 6.00E-58 | 0.47 |
