# Supplementary figures and images for: Chemical Compositions of Propolis from China and the United States and their Antimicrobial Activities Against Penicillium notatum
Source: Molecules. 2019 Oct 4;24(19):3576. doi: 10.3390/molecules24193576 (PMC6803850; doi:10.3390/molecules24193576)

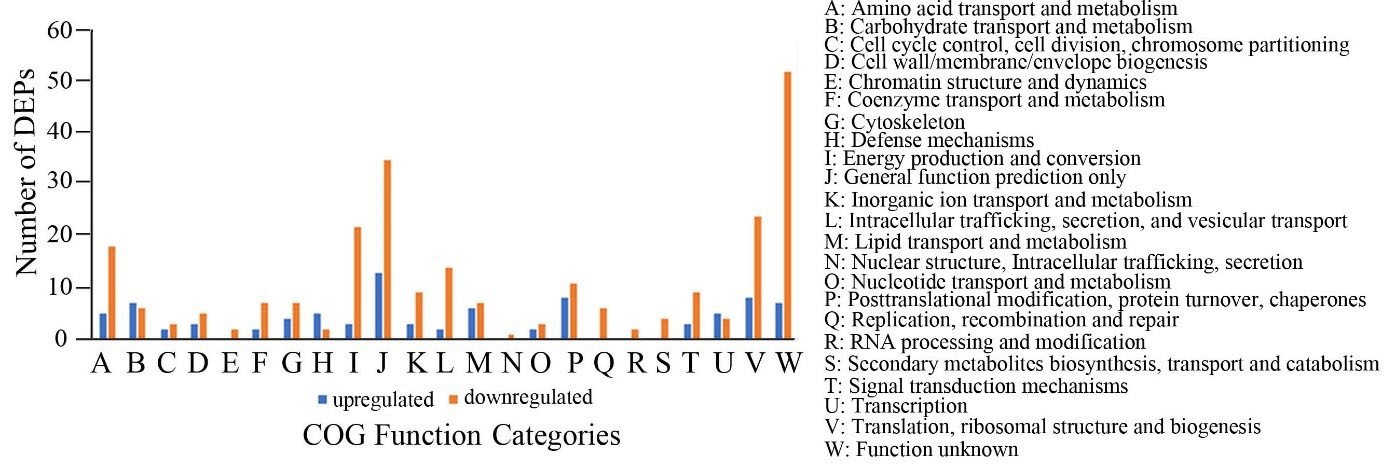

Supplement: Supplementary file 1 [file molecules-24-03576-s001.zip › Supplementary figure 1.jpg]
